# Winter Cherry

**Published:** 1865-02

**Authors:** 


*Winter Cherry.*—The berries of this plant, (*Physalis Alkekengi,*) cultivated in our gardens, have been successfully used by Dr. Saville, of London, in the treatment of obstinate gout. The berries are said to be most effective when allowed to ripen and begin to dry on the stem. From six to twelve berries, or an ounce of the expressed juice, may be taken for a dose; and much larger quantities are not injurious. They are consumed to a considerable extent in some parts of Europe as food. They are said to possess strong diuretic properties, and have been employed in suppression of urine, gravel and other complaints of the urinary passages.

